# Eligibility of monoclonal antibody-based therapy for patients with severe asthma: a Canadian cross-sectional perspective

**DOI:** 10.1186/s13223-018-0301-6

**Published:** 2018-11-22

**Authors:** Samira Jeimy, Michael William Tsoulis, Julie Hachey, Harold Kim

**Affiliations:** 1 Division of Clinical Immunology and Allergy, Department of Medicine, Western University, London, ON Canada; 2 School of Medicine, Stony Brook University, Stony Brook, NY USA; 3 Department of Medicine, Western University, London, ON Canada; 4 Division of Clinical Immunology and Allergy, St. Joseph’s Health Care London, Room B3-110, 268 Grosvenor Street, London, ON N6A 4V2 Canada

**Keywords:** Asthma, Biologics, Endotypes, Epidemiology

## Abstract

**Background:**

Based on immunologic phenotypes underlying asthma, use of monoclonal antibody based therapies is becoming the new standard of care for severe, corticosteroid refractory clinical symptoms. Patients may qualify for one or more of these targeted treatments, based on clinical characteristics and approved indications. However, the statistics are not well characterized, particularly in the Canadian population.

**Methods:**

The objective of this observational study was to identify and describe the proportion of patients with severe asthma who were eligible for targeting IgE, IL-5, or both pathways of immunomodulation. We reviewed a cross-sectional cohort of patients in a Canadian Allergy and Immunology referral practice. We also compared demographic and clinical characteristics of each group.

**Results:**

Of the 128 patients with severe asthma, 84 (66%) were eligible for omalizumab, 100 (78%) for mepolizumab, 52 (41%) for reslizumab, and 68 (53%) for benralizumab. Overlap in treatment eligibility varied; 68 (53%) patients were eligible for both omalizumab and mepolizumab, 47 (37%) were eligible for omalizumab and benralizumab, and 37 (29%) were eligible for all four medications. Patient demographics and clinical characteristics were similar, and levels of serum biomarkers varied based on locally approved prescribing criteria.

**Conclusion:**

In this severe asthma population from a Canadian Allergist’s practice, one-third of individuals qualified for all currently available biologics. 41–78% were eligible for at least one mAb. Patients were most likely to be eligible for mepolizumab. Objective assessments to determine asthma phenotype, along with further characterization of safety profiles will lead to further advances in asthma management.

## Introduction

Severe asthma is defined as asthma with poor symptom control, frequent severe exacerbations requiring systemic corticosteroids, and minor improvement in airflow limitation with bronchodilator therapy, or controlled asthma that worsens on corticosteroid taper [1]. 5–25% of individuals worldwide have severe asthma; however, the entity has major impact, with significant reduction in health-related quality of life [1], and utilization of 80% of asthma-related resources and health care costs ($8 billion/year combined for the United States, Australia, and Europe) [2]. Several immunologic phenotypes of asthma have been described, based on clinical and biochemical features. Allergic asthma is characterized by high levels of immunoglobulin (Ig) E, elevated fraction of exhaled nitric oxide (FeNO), and eosinophilic inflammation, whereas eosinophilic asthma involves eosinophilic inflammation, elevated FeNO, and recurrent asthma exacerbations [1].

Based on the different immunologic mechanisms underlying asthma, monoclonal antibody-based therapies have emerged as promising therapy for severe, corticosteroid refractory asthma. These include omalizumab (Genentech, Inc., South San Francisco, CA), which neutralizes IgE, mepolizumab (GlaxoSmithKline, Research Triangle Park, NC) and reslizumab (Teva, Jerusalem, Israel), which disrupt IL-5 signaling to reduce peripheral blood and pulmonary eosinophil counts, and benralizumab (AstraZeneca, Cambridge, UK), which binds to the α-chain of the interleukin (IL)-5 receptor on eosinophils to deplete eosinophils via antibody-dependent, cell-mediated cytotoxicity. Based on clinical characteristics and approved indications for use, patients may qualify for one or both of these targeted treatment options, although the proportions of individuals are not well characterized, particularly in the Canadian population.

The objective of this observational study was to identify and describe patients with severe asthma (as defined by International European Respirology Society (ERS)/American Thoracic Society (ATS) guidelines [1]), who are eligible for biologic therapy, and identify the proportion of patients eligible for targeting IgE, IL-5, or both pathways of immunomodulation. Individuals assessed were in a cross-sectional cohort of patients in a Canadian community Allergy and Immunology practice. We also compared demographic and clinical characteristics between cohorts of patients eligible for treatment with each biologic therapy.

## Methods

We performed a cross-sectional, retrospective chart review of patients with severe asthma, selected from a Canadian Allergy and Immunology referral practice for severe asthma in southwestern Ontario. We reviewed the entirety of the Allergist’s practice in order to reflect a real-world Canadian population. Patients were ≥ 12 years of age, with severe asthma defined according to ERS/ATS guidelines [1]. No exclusion criteria were applied once the individuals with severe asthma were selected for the study.

We documented patient demographics (age, gender, medical comorbidities), asthma history (control, medications, asthma-related healthcare resource utilization), and objective lung function assessments (spirometry as per ATS recommendations). We also documented serum biomarkers, including total serum IgE and blood eosinophil counts. A standardized chart abstraction form was used, to ensure systematic collection of data. The chart reviewer was blinded to the primary outcome of the study.

The primary outcome was the percentage of patients with severe asthma eligible for ≥ 1 biologic treatment. Eligibility was determined by regulatory labels (summarized in Table 1). Of note, Canadian indications for benralizumab (i.e., add-on maintenance treatment of adult patients with severe eosinophilic asthma) are more liberal than those used in clinical trials for this biologic.

**Table 1:** Eligibility criteria for biologic therapies

Criteria	Therapy			
	Omalizumab	Mepolizumab	Reslizumab	Benralizumab
Age (years)	≥ 6	> 18	> 18	> 18
Eosinophil count/µL and clinical criteria	N/A	≥ 150 or ≥ 300 in the previous 12 months AND inadequately controlled with high-dose ICS and an additional asthma controller (e.g., LABA)	≥ 400 AND inadequately controlled with medium-to-high-dose ICS and an additional asthma controller (e.g., LABA)	≥ 300 AND ≥ 2 asthma exacerbations in previous 12 months, OR ≥ 150 AND ≥ 1 asthma exacerbations in previous 12 months AND chronic OCS
Baseline IgE (kU/L)	≥ 30 and ≤ 700	N/A	N/A	N/A
Asthma characteristics	Moderate-severe symptoms inadequately controlled with ICS	Inadequate control with high dose ICS and additional controller(s) (e.g., LABA)	Inadequate control with high dose ICS and additional controller(s) (e.g., LABA)	Severe symptoms inadequately controlled with high-dose ICS and additional controller(s) (e.g., LABA)
Allergy	Evidence of sensitization with perennial aeroallergen with skin prick test or serum specific IgE	N/A	N/A	N/A

*ICS* inhaled corticosteroids, *LABA*, long-acting β_2_-adrenergic-receptor agonist, *OCS* oral corticosteroids

The study was conducted in accordance with the Declaration of Helsinki ethical practices. Ethics approval was obtained from the McMaster University institutional review board.

Statistical analyses were performed with Graph Pad Prism (Version 5, La Jolla, CA). The χ^2^ test for Independence or Fisher’s exact test for Independence, where appropriate, was used to analyze associations between two categorical variables. One-way ANOVA with Tukey’s multiple comparison test was used to compare mean scores of continuous data between categorical groups, namely omalizumab, mepolizumab, reslizumab, and benralizumab-eligible patients. If normality failed, Kruskal–Wallis with Dunn’s multiple comparison test was used. All tests were two-sided and p-values < 0.05 were considered statistically significant.

## Results

**Fig. 1:**
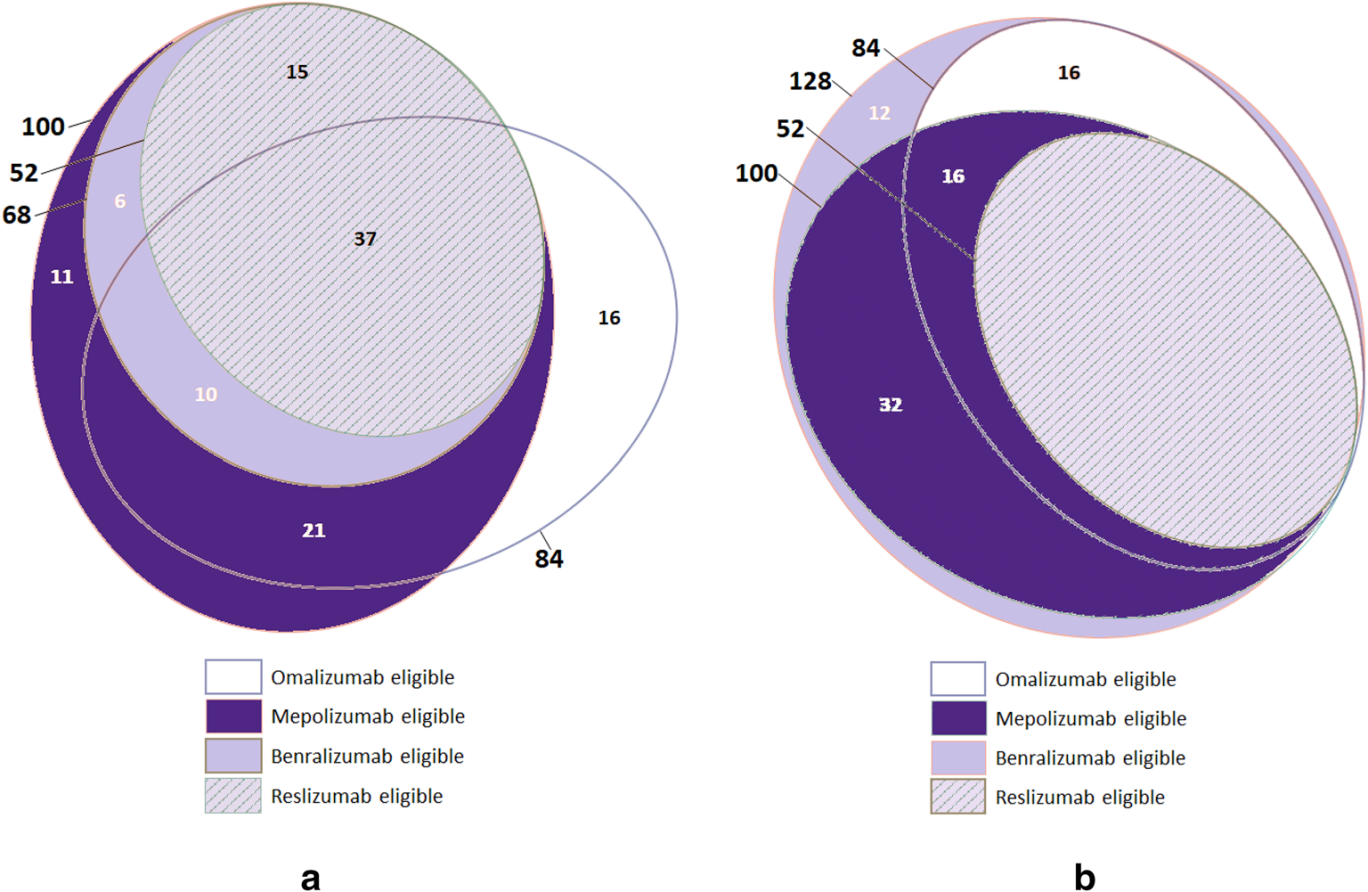
Overlap of patients eligible for biologic therapies, based on 128 patients with severe asthma from a Canadian Allergist and Immunologist’s referral practice, using clinical trial criteria (**a**) and current Canadian indications (**b**)

Of the 192 patients screened, 128 had severe asthma. Of these patients, 84 (66%) were eligible for omalizumab, 100 (78%) for mepolizumab, 52 (41%) for reslizumab, and 68 (53%) for benralizumab (Fig. 1). Overlap in treatment eligibility varied; using respective clinical trial criteria, 68 (53%) patients were eligible for both omalizumab and mepolizumab, 52 (41%) were eligible for mepolizumab and reslizumab, 47 (37%) were eligible for omalizumab and benralizumab, 68 (53%) were eligible for mepolizumab and benralizumab, 52 (41%) were eligible for reslizumab and benralizumab, and 37 (29%) were eligible for all four medications (Fig. 1a). Eleven patients were not eligible for any biologic; seven had IgE ≥ 700 kU/L with eosinophil count ≤ 150/μL, and four had IgE ≤ 30 kU/L and an eosinophil count ≤ 150/μL. Even in a population referred to an Allergist’s practice, there is thus a proportion of individuals with severe asthma who could benefit from further characterization of asthma endotype for personalized therapy.

Patient baseline demographics were similar between the four biologic-eligible groups (Table 2). The mean age for each group was 53.6, reflecting the large number of adults seen in the Allergy and Immunology practice. There was no significant difference in age between groups (p = 0.93). There were higher numbers of female patients in the total cross-section (Table 2), with a similar percentage within each biologic-eligible group (p = 0.95).

**Table 2:** Clinical characteristics of patients eligible for each biologic therapy

	Eligibility				
	All	Omalizumab	Mepolizumab	Reslizumab	Benralizumab
Demographic characteristic	(n = 128)	(n = 84)	(n = 100)	(n = 52)	(n = 68)
Age, years, mean (range)	53.26 (20–89)	53.07 (20–89)	53.87 (20–89)	54.79 (20–89)	53.49 (20–89)
Gender, female, *n* (%)	86 (67)	57 (68)	67 (67)	33 (63)	46 (68)
Current inhaled corticosteroid use, *n* (%)	125 (98)	82 (98)	98 (98)	52 (100)	68 (100)
ED visit(s)/hospitalization(s)^a^, *n*	52	35	38	24	29
% predicted FEV1, mean (SD)	70 (19)	73 (18)	67 (18)	70 (18)	69 (17)
≥ 150 eo/µL, *n* (%)	100 (78)	68 (81)	100 (100)	52 (100)	68 (100)
> 300 eo/µL, *n* (%)	68 (53)	47 (56)	68 (68)	52 (100)	68 (100)
≥ 400 eo/µL, *n* (%)	52 (41)	37 (44)	52 (52)	52 (100)	52 (76)
Total IgE, kU/L, mean (SD)	759 (1522)	231 (176)	753 (1486)	914 (1814)	932 (1723)

*ICS* inhaled corticosteroids, *ED* emergency department, *eo* eosinophils

^a^ED visits/hospitalizations for asthma-related illnesses in year prior to biologics

Clinical characteristics pertaining to asthma were also similar between groups. The majority of patients had controlled asthma according to the assessment of their symptom control, oral corticosteroid use, and exacerbation history (Table 2). Objective lung function assessments, indicated by the percent (%) of predicted FEV1, was not significantly different between groups (Table 2; p = 0.48).

Serum biomarkers varied between groups, related to the variations in eligibility criteria for each biologic therapy. There was a statistically significant difference in total serum IgE between eligibility groups (p < 0.001). Omalizumab eligible patients had significantly lower total serum IgE levels compared to each of mepolizumab, reslizumab and benralizumab-eligible patient groups (p < 0.05). These results reflect the upper limit of IgE (≤ 700 kU/L) associated with omalizumab eligibility. As a result of the eligibility criteria, mean blood eosinophil counts were higher in mepolizumab, reslizumab, and benralizumab-eligible patients, compared with omalizumab-eligible patients (Table 2).

## Discussion

Recent advances in identification of asthma endotypes and immunomodulatory targets have led to paradigm shifts in management of asthma, and biologics are becoming the new standard of care for severe asthma [3]. In this cross-section of individuals with severe asthma in a Canadian Allergist’s practice, approximately one-third (29%) of all patients were eligible for all biologic therapies. Eligibility for mepolizumab was highest (78%), and greater than its anti-IL-5 counterparts benralizumab and reslizumab, although all three biologics target a similar allergic endotype of asthma. The latter was based on a higher eosinophil threshold for benralizumab and reslizumab eligibility. Of note, we used clinical trial criteria to determine benralizumab eligibility in our analyses for Table 2. As shown in Fig. 1b, all of our patients would be eligible for this biologic based on current Canadian indications.

Our study extends the findings of the IDEAL study (Identification and Description of sEvere Asthma patients in a cross-sectionaL study), an industry (GSK) sponsored observational cohort study, of biologic eligibility in 670 individuals with severe asthma [4]. Our study consisted of 128 Canadian patients from an Allergist’s practice, whereas the IDEAL study included 88 Canadian patients [5]. As well, IDEAL study patients were recruited from respirology, primary care, and allergy clinics, resulting in differences in patient demographics. Lastly, our analysis includes eligibility for benralizumab, which was not yet approved at the time of the IDEAL study. Our results had some important differences. In our study population, almost all individuals with severe asthma were eligible for, and started on, biologic therapy. This was in contrast to the IDEAL study, where a considerable proportion of patients (65–76%) were not eligible for any of the three biologic therapies [4] (due to not fulfilling criteria for having exacerbations requiring oral corticosteroids or healthcare resource utilization). Among the general population, this highlights the clear remaining unmet medical need in individuals with severe asthma, who can potentially benefit from Allergist evaluation and determination of asthma phenotype to direct therapy.

Compared to the IDEAL study, treatment eligibility for omalizumab (targeting the persistent eosinophilic asthma endotype) was lower than that for mepolizumab in our population [4]. In the IDEAL study, phase III trial criteria was used to define eligibility for the anti-IL-5 biologic therapies, which likely accounts for the differences in our results. As in our study, mean blood eosinophil counts were higher in mepolizumab-eligible and reslizumab-eligible patients, compared with omalizumab-eligible patients, a result of the eligibility criteria.

Among all individuals with asthma, there is 68% reported overlap of patients with eosinophilic and allergic endotypes, based on an eosinophil count cut-off of 150 cells/µL [6]. As such, we anticipated that there would be an overlap in the proportion of patients eligible for anti-IL-5 therapy and anti-IgE therapy. We found that 53% of patients were eligible for omalizumab and mepolizumab, and 37% were eligible for omalizumab and benralizumab. This is in contrast to the IDEAL study, where 27–37% of patients were eligible for both omalizumab and mepolizumab [4]. Again, the phase III criteria used to determine anti-IL-5 eligibility may have affected the results of the IDEAL study. As well, our patients were from an Allergist’s referral base, resulting in a large proportion of individuals with allergic and eosinophilic asthma. The overlap between mepolizumab and reslizumab was 41%, although both drugs target the same immunologic pathway by inhibiting IL-5. This was driven by the different thresholds of eosinophils applied in the eligibility criteria, with the 400 cells/µL criterion for reslizumab being the limiting factor with the greatest impact on eligibility.

Our study has some important limitations. Firstly, we performed retrospective chart reviews, which is subject to constraints and biases related to data availability. To mitigate this limitation, we ensured systematic chart abstraction using a standardized data collection form, and used explicitly stated, universally accepted (ERS/ATS) criteria to define our variables. Secondly, our objective in this study was to study a cross-section of Canadians with severe asthma, which limits generalizability to the general population worldwide. Thirdly, our sample was derived from an Allergist’s referral base, resulting in a larger proportion of individuals with eosinophilic or allergic endotypes of asthma.

Our study identified that a large proportion of individuals with severe asthma could benefit from characterization of asthma endotype, to drive targeted therapy. Based on the overlap of the allergic and eosinophilic asthma endotypes, we noted overlap in eligibility for the different mechanisms of action of currently available biologic therapies. Careful clinical characterization of asthma control and biomarkers will hopefully result in further personalization and optimization of asthma management.

## Abbreviations

mAb: monoclonal antibody
IL: interleukin
FeNO: fraction of exhaled nitric oxide
IgE: immunoglobulin E
ERS: European Respirology Society
ATS: American Thoracic Society
ANOVA: analysis of variance
FEV1: forced expiratory volume in one second
IDEAL: Identification and Description of sEvere Asthma patients in a cross-sectionaL study
ICS: inhaled corticosteroids
LABA: long-acting β_2_-adrenergic-receptor agonist
OCS: oral corticosteroids
ED: emergency department
Eo: eosinophils
